# Korean national athletes’ knowledge, practices, and attitudes of doping: a cross-sectional study

**DOI:** 10.1186/s13011-017-0092-7

**Published:** 2017-02-14

**Authors:** Taegyu Kim, Young Hoon Kim

**Affiliations:** 1 Department of Sports Medicine and Science, Taereung National Training Center of the Korean Olympic Committee, 727, Hwarang-ro, Nowon-gu, Seoul 01794 Republic of Korea; 2 Department of Medicine, Graduate School of Jeju National University, 102 Jejudaehak-ro, Jeju-si, Jeju-do 63243 Republic of Korea; 3 Mac Rehabilitation Clinic, 386, Nohyeong-ro, Jeju-si, Jeju-do 63083 Republic of Korea

**Keywords:** Adult, Adolescent, Performance enhancement attitude scale, Anti-doping

## Abstract

**Background:**

Despite the efforts of the World Anti-Doping Agency and national anti-doping agencies at the international level, a relatively low and steady rate of positive doping tests still persists all over the world. Evidence on adolescents using doping substances exists, and the proportion of adolescents engaging in doping practices is small but significant. In relation to the international research trends on anti-doping, this study aims to evaluate doping knowledge, practices, and attitudes among Korean adult and adolescent elite athletes to provide effective information on anti-doping policies and education programs.

**Methods:**

This study was a cross-sectional study of 454 Korean elite athletes (249 adults in 23 events and 205 adolescents in 22 events). Data were collected by an interviewer-administered questionnaire containing items regarding doping practices and knowledge, brief definitions of performance-enhancing substances/methods and recreational substances, and the Performance Enhancement Attitude Scale (PEAS).

**Results:**

Adolescent (47.3%) and adult (57.0%) athletes received information on banned substances of their respective sports from the Korea Anti-Doping Agency, and 39.0 and 53.4% of adolescents and adults, respectively, had knowledge of banned substances and had permissive attitudes toward doping compared to those who were unaware. Adolescent and adult athletes have inadvertently (1.5 and 3.6%, respectively) or knowingly (1.0 and 2.8%, respectively) taken banned performance-enhancing substances, and 2.4 and 3.2%, respectively, knew someone who had taken banned substances. And the adolescent athletes in motor skill category (PEAS: 40.24 ± 10.91) were more permissive toward doping than those in team category (PEAS: 35.08 ± 10.21).

**Conclusion:**

An in-depth anti-doping education for Korean athletes should be more widely implemented, and effective anti-doping policy should meet the athletes’ demographic characteristics, personalities, and values.

## Background

The use of performance-enhancing substances in sports is not a new phenomenon [1]. To improve their performance, over 5,000 years ago, ephedra was used in China, and athletes used stimulants such as dried figs, mushrooms, and strychnine during the Ancient Greek Olympic Games [2–4]. It is conservative to say that the use of performance-enhance in substances occurred naturally through human history [5]. At present, the World Anti-Doping Agency (WADA) defined doping as the use of illegal performance-enhancing drugs and methods to improve performance [6, 7].

In 1928, performance-enhancing drugs were first banned by the International Amateur Athletics Federation (now the International Association of Athletics Federations) [8]. When two deaths caused by amphetamine occurred in 1960 and 1967, the International Olympic Committee (IOC) formed the Medical Commission and tested for banned substances in 1967 and 1968, respectively [8]. For effective anti-doping activities, the IOC found the need for cooperation between sports authorities and the government, and established the WADA in 1999 [8]. The WADA publishes a list of prohibited substances and methods annually and tests the blood and/or urine of athletes who registered in the National Olympic Committee, randomly or systemically, for doping evidence [9]. Moreover, the WADA and national anti-doping agencies educate all athletes to foster abstinence from banned performance-enhancing substances [8, 9].

With all these efforts at the international level, the rate of positive doping tests is around 2% [6]. A previous study mentioned that this relatively low and steady rate is not a major problem in sport if it appears that the prevalence rate of alternative sources is considerably higher [6]. However, it reported that more than 30% of competitive elite athletes have used at least one substances for enhancing their performance [10] and the percentage of unconfirmed doping cases is thought to be significantly higher [11]. Moreover, evidence on adolescents using doping substances exists, and the proportion of adolescents engaging in doping practices is small but significant [1]. Stilger & Yesalis [12] found that American high school football players use anabolic androgenic steroids starting at an average age of 14 years, and Calfee & Fadale [3] mentioned that steroid use prevalence in high school ranged from 4 to 11% in boys and up to 3.3% in girls. The increase of the use of substances to enhance sports-performance among adolescents and even pre-adolescents is a worrying trend because of the relatively unknown associated risks [13, 14]. Because of not only the relative difficulty in determining all banned substances utilized by elite athletes, but also the more sophisticated doping methods of athletes who used them to enhance sports performance and their intent to evade testers [1, 15], the identification of driving forces behind the doping behaviors of athletes was required to develop effective anti-doping programs [16]. Actually, Smith and Stewart (2009) argued that it is doubtful that WADA’s anti-doping policy is effective in maintaining a level playing field, or is the best means of protecting the health of athletes [17].

Various factors, such as representations, knowledge, attitudes, personality, and motivation, are a great influence on the transformation of normative to deviant behaviors [18]. This option has implied the need to evaluate factors for predicting and identifying the doping behavior in elite athletes [18]. Moreover, a recent meta-analysis revealed that positive attitudes toward doping are strong positive correlates of doping intensions and behaviors [19]. Therefore, to develop targeted anti-doping policies and programs, understanding an athlete’s knowledge of, attitudes toward, and practices in doping is crucial [20].

The Korea Anti-Doping Agency (KADA) conducted doping control tests for 3,782 Korean elite athletes, and found seven anti-doping rule violations, with the exception of 28 cases in bodybuilding, which has a different standard of competition from other sports events, were reported in 2015 [21]. Despite 140 anti-doping education programs with 12,272 participants, including the athletes in 2014, a small but steady number of violations exists [22]. Thus, in relation to the international research trends on anti-doping, this study aims to evaluate the doping knowledge, practices, and attitudes among Korean adult and adolescent elite athletes and compare doping attitudes based on doping knowledge and practices, gender, and sports event categories to provide effective information on anti-doping policies and education programs.

## Methods

### Participants

A descriptive cross-sectional research design was used to identify the doping knowledge, practices, and attitudes of adult and adolescent elite athletes in Korea. A total of 468 (257 adults in 23 events and 211 adolescents in 22 events) of athletes enrolled in the Korean National Team in the 2013 and 2014 seasons were selected for this study based on their participation in international multi-sports events. The participants were divided into adult (>18 years old) and adolescent (≤18 years old) athletes. Adult athletes gave their informed consent to participate in this study, while parental consent was obtained for adolescent athletes. The participants completed a self-report questionnaire regarding their demographic information, events, and years of their career, and their events were categorized as follows [23]: speed and power, endurance, motor skills, and team. For some events whose categories was ambiguous, a high-performance coach of each discipline was consulted and asked to select the categories [24]. The speed and power category contained athletics, weightlifting, taekwondo, judo and so on, and the endurance category, swimming and cycling; the motor skill category, tennis, fencing, badminton, shooting, golf, etc.; and the team category, handball, rugby, hockey, soccer, volleyball, basketball, etc. Fourteen questionnaires were excluded for inappropriate responses; thus, a total of 454 responses (249 adults in 23 events and 205 adolescents in 22 events) were used in data analysis. Table 1 shows the participants’ characteristics.

**Table 1:** Participants’ Characteristics

	N (%)	Gender N (%)		Age (years)	Career (years)	PEAS
		Male	Female			
Adolescent athletes	205 (45.2)	116 (38.7)	89 (57.8)	16.75 ± 1.45	6.82 ± 2.61	37.66 ± 11.20
Adult athletes	249 (54.8)	184 (61.3)	65 (42.2)	24.05 ± 3.53***	12.65 ± 3.89***	40.22 ± 13.95*
Total athletes	454 (100)	300 (100)	154 (100)	20.76 ± 4.59	10.02 ± 4.45	39.07 ± 12.83

All data were expressed as mean and standard deviation or frequencies and percentages

*PEAS* Performance Enhancement Attitude Scale

*p < 0.05 and ***p < 0.001: tested by the independent *t*-test between adolescent and adult athletes

Statistics were shown as follows: *t*_411.441_ = 3.888, *p* < 0.001 in the Age variable; *t*_435.450_ = −18.337, *p* < 0.001 in the Career variable; and *t*_451.735_ = −2.170, *p* = 0.03 in the PEAS variable

### Data collection procedure

Data was collected by an interviewer-administered questionnaire for receiving higher response rates compared to self-administered [25] and for helping respondents better understand each question. For minimizing the social desirability bias produced by the interaction between respondent and interviewer, all interviewers were carefully trained and monitored by the authors [25]. The questionnaire contained items regarding the doping practices and doping knowledge, brief definitions of performance-enhancing substances/methods and recreational substances, the Performance Enhancement Attitude Scale (PEAS), and a cover letter explaining the purpose of this study [24, 26]. All participants were interviewed individually, and their records were strictly confidential.

Doping knowledge and practices were identified using the five questions which were proposed by Moran et al. [24] and were translated into Korean by Kim and Kim [27]. Two separate questions on whether they want to know or receive information on the banned substances, wherein participants were asked to choose the responses (i.e., yes or no), and were used to identify their doping knowledge. The questionnaire contained the list of banned performance-enhancing substances and methods; however, it was not shown to the participants until the question involved the list. Moreover, the doping practices were identified by three questions on the current use of and experience with performance-enhancing substances. Participants who selected “yes” were required to answer the follow-up question on the type of substance.

Doping attitude was defined as an individual’s predisposition toward the use of banned performance-enhancing substances and methods [20] which is quantitatively measured by the PEAS questionnaire which was proposed by Petroczi [26] and was translated into Korean by Kim and Kim [27]. The PEAS consisted of 17 items on a six-point Likert-type scale (strongly disagree (1), strongly agree (6), and no neutral, middle point), and all 17 items were scored in the same direction. The total score ranged from 17 to 102, and the theoretical middle-point was 59.5 [28]. A high score means a permissive attitude toward doping, while a low score denotes an intolerant attitude [26]. According to previous studies, the Cronbach’s alpha values for PEAS range from 0.71 to 0.91 [20, 24, 28], and in this study, the Cronbach’s alpha values were 0.85. In 2014, Kim and Kang [29] mentioned that the 9-item Korean PEAS questionnaire had more validity than the 17-item for Korean athletes. However, in this study, *χ* ^2^ per degree of freedom (*χ* ^2^/df) and Root Mean Square Error of Approximation (RMSEA) values indicated an acceptable fit for the 17-item (*χ* ^2^/df = 2.98, RMSEA = 0.070) compared to the 9-item (*χ* ^2^/df = 5.23, RMSEA = 0.097), although both Tucker Lewis Index (TLI) and Comparative Fit Index (CFI) of the 17-item (TLI = 0.764, CFI = 0.792) and the 9-item (TLI = 0.801, CFI = 0.845) were just below 0.9 and were not acceptable. Therefore, the 17-item PEAS questionnaire was used in this study.

### Statistical analyses

To assess factor structure, a confirmatory factor analysis was done through AMOS 20.0 (2011 Amos Development Corporation), and the evaluated model was fit with the following parameters: *χ* ^2^/df (acceptable when < 3.00); RMSEA (close fit ≤ 0.05, reasonable fit ≤ 0.08); TLI and CFI (both acceptable when ≥ 0.90, good ≥ 0.95). All statistical analyses were performed using SPSS version 20.0 for Window (SPSS Inc., Chicago, IL, USA), and all data was expressed as mean and standard deviation or frequencies and percentages, depending on the characteristics of the variables after assessing the normality of the data. An independent *t*-test was used to compare the PEAS score in terms of gender or responses of the athletes selected for questions on doping knowledge and practices. A one-way analysis of variance with a post hoc least significance difference (LSD) test was used to analyze the PEAS score with regard to sports event categories. Statistical significance was identified at *p* < 0.05.

## Results

### Doping knowledge and attitudes

Table 2 showed the knowledge of doping and the attitudes depending on the response in adolescent and adult athletes. For the question on whether they have received information about banned substances in their sport, 47.3% of adolescent athletes and 57.0% of adult athletes responded ‘yes’. The adolescent athletes with the positive response (PEAS score: 39.37 ± 11.06) had more a permissive attitude toward doping than athletes with the negative response (PEAS score: 36.13 ± 11.16) (*t*_203_ = 2.085, *p* = 0.03). The adult athletes with a positive response (PEAS score: 41.77 ± 13.60) had more a generous attitude than those who responded “no” (PEAS score: 38.17 ± 14.20) (*t*_247_ = 2.032, *p* = 0.04). For the question on whether they knew exactly which substances were banned, 39.0 and 53.4% of adolescent and adult athletes, respectively, replied in the affirmative. The attitude toward doping of adolescent athletes who responded “yes” (PEAS score: 39.74 ± 10.92) was more affirmative than that of those who responded “no” (PEAS score: 36.34 ± 11.22) (*t*_203_ = 2.139, *p* = 0.03). The adult athletes who replied in the affirmative (PEAS score: 42.10 ± 13.90) had a more permissive attitude toward doping than those who responded “no” (PEAS score: 37.24 ± 13.58) (*t*_247_ = 2.709, *p* = 0.007).

**Table 2:** Doping Knowledge and Attitudes

Response		Yes	No	*t* (*p*)
Question: Have you received information about banned substances in you sport?				
Adolescent athletes (*n* = 205)	N (%)	97 (47.3)	108 (52.7)	-
	PEAS	39.37 ± 11.06	36.13 ± 11.16	*t*_203_ = 2.085 *p* = 0.03
Adult athletes (*n* = 249)	N (%)	142 (57.0)	107 (43.0)	-
	PEAS	41.77 ± 13.60	38.17 ± 14.20	*t*_247_ = 2.032 *p* = 0.04
Question: Are you confident in your knowledge about banned substances in your sport?				
Adolescent athletes (*n* = 205)	N (%)	80 (39.0)	125 (61.0)	-
	PEAS	39.74 ± 10.92	36.34 ± 11.22	*t*_203_ = 2.139 *p* = 0.03
Adult athletes (*n* = 249)	N (%)	133 (53.4)	116 (46.6)	-
	PEAS	42.10 ± 13.90	37.24 ± 13.58	*t*_247_ = 2.709 *p* = 0.007

All data were expressed as mean and standard deviation or frequencies and percentages

*PEAS* Performance Enhancement Attitude Scale

Table 3 showed the sources of information about banned performance-enhancing substances. About two-threes of adolescent athletes with a positive response on whether they have received information about banned substances in their sport received the information on banned substances from KADA (63.9%), the coach (16.5%), and medical support (12.4%). A total of 81.7% of adult athletes who responded “yes” to the same question received the information from KADA.

**Table 3:** Sources of Information about Banned Performance-Enhancing Substances

*N* (%)	KADA	Coach	Medical support	Fellow competitor	Parents	Others
Adolescent athletes (*n* = 97)	62 (63.9)	16 (16.5)	12 (12.4)	3 (3.1)	2 (2.1)	2 (2.1)
Adult athletes (*n* = 142)	116 (81.7)	8 (5.6)	6 (4.2)	8 (5.6)	0 (0)	4 (2.8)

All data were expressed as frequencies and percentages

*KADA* Korea Anti-doping Agency

### Doping practices and attitudes

Table 4 shows the doping practices and attitudes based on the adolescent and adult athletes’ responses For the question on whether they inadvertently took any banned substance, 1.5 and 3.6% of the adolescent and adult athletes, respectively, responded “yes”; of the 12 athletes, nine have used recreational substances and three took performance-enhancing substances. The attitude toward doping of adults with positive response (PEAS score: 53.22 ± 7.45) was significantly more permissive than that of athletes who responded ‘no’ (PEAS score: 39.74 ± 13.91) (*t*_10.225_ = 5.109, *p* < 0.001). For the question on whether they had knowingly taken any banned performance-enhancing substances, 1.0 and 2.8% of the adolescent and adult athletes have knowingly taken any banned performance-enhancing substances, and eight out of a total of 9 athletes have taken recreational substances. The adults who took any banned substance (PEAS score: 57.14 ± 10.06) had a more generous attitude toward doping compared to adult athletes who responded ‘no’ (PEAS score: 39.74 ± 13.75) (*t*_247_ = 3.320, *p* = 0.001). For the question on whether they knew someone who experienced taking banned substances, 2.4 and 3.2% of the adolescent and adult athletes, respectively, responded “yes”; of the 13 athletes, seven have taken recreational substances while the others took banned performance-enhancing substances. The attitude toward doping of the adults (PEAS score: 53.88 ± 6.92) was more affirmative than that of adult athletes who responded ‘no’ (PEAS score: 39.77 ± 13.90) (*t*_8.999_ = 5.416, *p* < 0.001).

**Table 4:** Doping Practices and Attitudes

Response		Yes	No	*t* (*p*)
Question: Have you ever inadvertently taken any substances whose use is prohibited in your sport?				
Adolescent athletes (*n* = 205)	N (%)	3 (1.5)	202 (98.5)	
	PEAS	42.33 ± 15.18	37.59 ± 11.17	*t*_203_ = 0.726 *p* = 0.46
Adult athletes (*n* = 249)	N (%)	9 (3.6)	240 (96.4)	
	PEAS	53.22 ± 7.45	39.74 ± 13.91	*t*_10.225_ = 5.109 *p* < 0.001
Question: Have you ever knowingly taken any substances whose use is prohibited in your sport?				
Adolescent athletes (*n* = 205)	N (%)	2 (1.0)	203 (99.0)	
	PEAS	35.5 ± 13.44	37.68 ± 11.22	*t*_203_ = −0.274 *p* = 0.78
Adult athletes (*n* = 249)	N (%)	7 (2.8)	242 (97.2)	
	PEAS	57.14 ± 10.06	39.74 ± 13.75	*t*_247_ = 3.320 *p* = 0.001
Question: Do you personally know any athletes who are taking, or have previously taken, prohibited substances?				
Adolescent athletes (*n* = 205)	N (%)	5 (2.4)	200 (97.6)	
	PEAS	42.60 ± 10.07	37.54 ± 11.23	*t*_203_ = 0.998 *p* = 0.32
Adult athletes (*n* = 249)	N (%)	8 (3.2)	241 (96.8)	
	PEAS	53.88 ± 6.92	39.77 ± 13.90	*t*_8.999_ = 5.416 *p* < 0.001

All data were expressed as mean and standard deviation or frequencies and percentages

*PEAS* Performance Enhancement Attitude Scale

### Doping attitudes depending on gender and sports event categories

Table 5 showed the attitudes of doping depending on gender and sports event categories. In adolescent athletes, the difference of PEAS was not significant between males and females (*t*_203_ = −1.018, *p* = 0.31), but the athletes for the motor skill category (PEAS score: 40.24 ± 10.91) had a more generous attitude toward doping compared to the team category (PEAS score: 35.08 ± 10.21) (*F*_(3, 201)_ = 2.740, *p* = 0.04). In adult athletes, no significant differences between genders (*t*_247_ = −0.603, *p* = 0.54) and among the sports event categories were found (*F*_(3, 245)_ = 2.363, *p* = 0.07).

**Table 5:** Doping Attitudes based on Gender and Sports Event Categories

	Variables		*N* (%)	PEAS	*t* (*p*)/*F* (*p*)
Adolescent athletes (*n* = 205)	Gender	Male	116 (56.6)	36.97 ± 11.48	*t*_203_ = −1.018 *p* = 0.31
		Female	89 (43.4)	38.57 ± 10.83	
	Sports event categories	Speed & power	43 (21.0)	39.21 ± 10.92	*F*_(3, 201)_ = 2.740 *p* = 0.04
		Endurance	29 (14.1)	36.90 ± 13.47	
		Motor skill	58 (28.3)	40.24 ± 10.91*	
		Team	75 (36.6)	35.08 ± 10.21	
Adult athletes (*n* = 249)	Gender	Male	184 (73.9)	39.91 ± 14.67	*t*_247_ = −0.603 *p* = 0.54
		Female	65 (26.1)	41.12 ± 11.75	
	Sports event categories	Speed & power	60 (24.1)	44.25 ± 14.25	*F*_(3, 245)_ = 2.363 *p* = 0.07
		Endurance	15 (6.0)	40.40 ± 14.63	
		Motor skill	93 (37.3)	39.28 ± 13.16	
		Team	81 (32.5)	38.30 ± 14.14	

All data were expressed as mean and standard deviation or frequencies and percentages

*PEAS* Performance Enhancement Attitude Scale

*p < 0.05: tested by the post hoc LSD test between motor skill and team in adolescent athletes

## Discussion

This cross-sectional study is to confirm the doping knowledge, practices, and attitudes in Korean adult and adolescent elite athletes, respectively and to compare their doping attitudes depending on knowledge and practices on doping, gender and sports event categories. This study revealed that approximately 50% of all athletes knew the banned performance-enhancing substances for their sports through KADA, and <50% of the athletes precisely knew their sports’ banned substances. Athletes who knew the banned substances of their respective sports had more permissive attitudes toward doping than those who were unaware. Only a few athletes admitted to have inadvertently or knowingly taken banned performance-enhancing substances in their sports or recreational substances, and the adult athletes were more positive toward doping. Moreover, adult athletes had a tendency to overestimate the prevalence of banned substances use, and they had more positive attitudes toward doping than those who did not. Adolescent athletes in the motor skill category were more permissive toward doping than those in the team category.

Doping has become a serious problem in 20^th^ century competitive sport, and it is a very complex phenomenon [30]. Thus, the WADA and national anti-doping agencies, including KADA, test the blood and/or urine for all elite athletes to detect evidence of doping and to educate them to encourage the abstinence from banned performance-enhancing substances [9]. Moran et al. [24] reported that 62.6% of athletes of various nationalities said that they had received information on banned substances in their sport, and 48.8% felt confident with their knowledge. Muwonge et al. [20] showed that two-thirds of Ugandan athletes replied in the affirmative to the question on whether they had received information regarding banned substances in their sport. In this study, 47.3% of adolescent athletes and 57.0% of adult athletes respond that they had received information on banned performance-enhancing substances, and 39.0 and 53.4% exactly knew what those banned substances were for adolescent and adult athletes, respectively. The continued efforts of the WADA and national anti-doping agencies resulted in the increasing awareness of athletes on anti-doping rules [31]; however, this study shows a low level of doping knowledge among athletes. Moreover, Fürhapter et al. [32] insisted that knowledge regarding the potential negative side effects of performance-enhancing substances is poor especially among adolescent athletes. These imply that a more widespread and in-depth anti-doping education is needed and that a more coherent, organized structure is essential, as suggested by Moran et al. [24].

As doping knowledge has influenced behavior on doping [18], an important factor for the development of efficient and sustainable preventive strategies for doping is an evaluation of the level of knowledge and attitudes with regard to doping in sports [32]. Thus, similar to Bradley et al. [33], we inferred that there was any relationship between doping knowledge and attitude and predicted that athletes with no information on the banned substances had a higher PEAS. However, this study shows that the PEAS scores of both adolescent and adult athletes who received information on specific banned substances were higher than those who did not; therefore, athletes who knew about banned performance-enhancing substances were more permissive toward doping. These results support the argument that the understanding of drug use and doping in sports remains limited in implementing an efficient prevention program [18] and the WADA’s statement that anti-doping research should include sociological studies of athletes’ attitudes and beliefs toward the use of banned substances in sports [31].

If guaranteed an Olympic medal win, 195 of 198 athletes who participated in the Lillehammer Olympic answered that they would be willing to take a banned performance-enhancing substance, and above 50% of them would also be willing to take a substance although it could lead to death for them [3]. In this case, it was known that many high-level athletes often approach their sport with a ‘win at all costs’ mentality including the permissive attitude of doping [3] and most of them trended to rationalize their doping behavior through the ‘false consensus effect’ [31]. Moran et al. [24] reported that 9.4 and 11% of athletes in various nationalities admitted that they inadvertently and knowingly, respectively, used banned substances, and Muwonge et al. [20] mentioned that 3.9% of Ugandan athletes had ever used the performance-enhancing substances, of which 3.3% admitted to recent use. Uvacsek et al. [34] showed that 14.6 and 31.7% acknowledged using banned substances and recreational substances, respectively. In this study, adolescent and adult athletes had inadvertently (1.5 and 3.6%, respectively) and knowingly (1.0 and 2.8%, respectively) taken banned substances. Furthermore, though there was a great difference in sample size between groups, the PEAS score of adult athletes with inadvertent use and knowledgeable experience of doping substances was higher than that of those without. These results correspond to those of Moran et al. [24] wherein some athletes who inadvertently or knowingly doped had a more positive attitude toward doping. Besides, this study shows that the adult athletes who knew someone who experienced doping had a more positive attitude toward doping, which in turn supports the references by Morente-Sanchez and Zabala [31] that the decision to take banned substances is influenced by the assumption that the competitors are also taking them. In other words, familiarity with banned substances through exposure to or observation of others’ doping practices may influence an athlete to ultimately decide to dope themselves [24]. Therefore, in relation to athletes’ personalities, an effective anti-doping program must include identification of athletes who are most vulnerable to doping [35], by checking the athletes’ values, such as a “win at all costs” mentality, and their familiarity with banned substances.

The use of banned substances differs according to the demand of a specific sport [31]. Team-based sports and sports requiring motor skills could be less influenced by doping practices than individual, self-paced sports [31]. Kondric et al. [8] reported that the absolute number of adverse or atypical analytical findings of athletes of track and field athletics, which is highly physically demanding, than in curling [9]. In this study, unlike the adults, the adolescent athletes in the motor category had a more tolerant attitude toward doping behavior compared with those in the team category. Thus, to develop an adequate anti-doping program, sports event characteristics and doping likelihood should be considered. Furthermore, Fürhapter et al. [32] showed that 2.5–5.3% of adolescents aged 13–19 years consumed banned substances such as anabolic steroids. Unsurprisingly, the negative and positive effects of banned substances were not known in the young population [36]. McNamee [14] also claimed that the harms of such substances in adolescents are relatively unknown. These alarming uncertainties necessitate an immediate intervention [32]. Therefore, a differential anti-doping policy for adolescent athletes from adults must be in place for eradicating the use of banned performance-enhancing substances.

Doping is affected by multidirectional factors in sports [30]. Thus, the current detection-based anti-doping policy does not automatically eradicate the use of banned performance-enhancing substances [16]. One of the key factors in designing effective anti-doping programs is the conceptual clarity of the psychological mechanisms that influence doping behavior [37]. Therefore, this study identified not only doping knowledge and practices but also attitudes toward doping among Korean national adolescent and adult athletes and compared doping attitudes based on doping knowledge and practices, gender, and sports event categories. Although the PEAS questionnaire was widely used to assess doping attitudes among adult and adolescent athletes [38], there were some conflicting evidences on the reliability and the validity of the PEAS [28]. In this study, both the 17-item and the 9-item Korean PEAS questionnaire did not provide an excellent fit. Furthermore, there was a lack of evidence to support the validity of the Korean PEAS questionnaire for either adult or adolescent athletes. Therefore, the full and/or short version Korean PEAS questionnaire was required to identify further validation for Korean adult and adolescent athletes. Also, as being exceedingly competitive or win-orientated is often explained in connection with doping behavior [39], further studies should include the correlation of psychosocial factors, i.e., sports orientation and the identification of changing attitudes toward doping, for effective anti-doping programs.

## Conclusion

Approximately 50% of Korean national adolescent and adult athletes knew the banned performance-enhancing substances for their respective sports; however, they had more permissive attitudes toward doping than athletes who have no knowledge of banned substances. Moreover, a few athletes have taken banned or the recreational substances and the adult athletes among them were more positive toward doping. The adult athletes who knew someone who had experience with taking banned substances had a more permissive attitudes to doping. Furthermore, the adolescent athletes in the motor skill category were more permissive toward doping than those in the team category. Therefore, an in-depth anti-doping education should be more widely implemented, and effective anti-doping policy should meet the athletes’ demographic characteristics, personalities, and values.

## Abbreviations

IOC: International olympic committee
KADA: Korea anti-doping agency
PEAS: Performance enhancement attitudes scale
WADA: World anti-doping agency
